# Quality and nutritional properties of noodles: a systematic comparison of noodles based on blue, purple, black, and white whole wheat flours

**DOI:** 10.3389/fnut.2025.1738148

**Published:** 2025-12-12

**Authors:** Yichen Li, Xiaomeng Yu, Xiaoling Tian, Mengqin Li, Jian Zhang, Yan Ma, Chenxi Yin

**Affiliations:** 1 College of Food Science and Technology, Henan Agricultural University, Zhengzhou, China; 2 Postdoctoral Station of Crop Science, Henan Agricultural University, Zhengzhou, China

**Keywords:** colored wheat, whole wheat flour, noodle quality, anthocyanin retention, mineral

## Abstract

**Background:**

The increasing demand for nutritionally enriched foods has driven interest in colored wheat varieties, which may offer enhanced nutritional benefits. This study aims to compare the quality of noodles made from whole flours of blue, purple, and black wheat with a white wheat control.

**Methods:**

Noodles were formulated using different substitution levels of colored whole wheat flours, and their quality was evaluated based on nutritional properties, texture, and antioxidant capacity.

**Results:**

The results showed that colored whole wheat flours significantly improved the nutritional properties compared to white wheat. Black wheat exhibited the highest selenium content (5.28 μg/g), while blue wheat had the highest anthocyanin level (14.15 mg/100 g). The incorporation of colored wheat flours increased moisture content (9.15–11.36%) and cooking loss (5.22–8.34%), while reducing noodle hardness, with the 75% purple wheat substitution showing the lowest value (5347.08 g). Although processing and cooking reduced anthocyanin content and ABTS radical scavenging activity, noodles made with colored wheat retained significantly higher levels of anthocyanins (42.75–88.97%) and antioxidant capacity (16.31–47.42%) than those made with white wheat.

**Discussion:**

These findings suggest that colored wheat varieties hold significant potential for developing functional noodles with improved nutritional profiles. Despite changes in texture, these noodles offer enhanced antioxidant activity without compromising their quality, making them a viable option for nutritionally enriched food products.

## 1 Introduction

As one of the three major cereals in the world, wheat is popular all over the world (1). With the transformation of food consumption to nutrition and health, the development of the functional properties of wheat staple foods has gained increasing attention (2). Nowadays, hidden hunger, a condition characterized by an imbalance in nutritional intake and a significant deficiency in certain nutrients, poses a huge challenge for the populace. This issue can be effectively addressed through the application of biological breeding techniques (3). In contrast to traditional wheat, colored wheat represents a distinctive cereal resource with higher concentrations of anthocyanins and antioxidant compounds, imparting enhanced nutritional properties compared to conventional wheat. Colored wheat has also demonstrated promising potential in terms of antioxidant, anti-tumor, and anti-inflammatory effects (4–6). Particularly, the majority of anthocyanins in wheat are found in the cortex, and different colored wheat has distinct anthocyanin kinds and places (7), most studies use whole wheat flour for food production to satisfy the desire for balanced nutrition and healthy diets (8, 9).

Current research on optimizing the processing of cereal-based foods often prioritizes baked goods such as bread, cakes, and biscuits, driven by the desirable sensory attributes imparted by baking (10–13); however, throughout the baking process, compounds including anthocyanins and lysine experience varying degrees of degradation (14–16). In contrast, noodles mainly cooked by boiling are usually in a gentler thermal regime, which has potential advantages in preserving the nutritional integrity and bioactive compounds of colored grain products.

Noodles were widely cherished by Asian consumers and boasted a broad market prospect, with approximately 40% of wheat in China being used for noodle production (17, 18). With the development of functional noodle formulations in recent years, we are no longer satisfied that refined wheat noodles can provide adequate protein and carbohydrates. The goal of using mixed grain flour in the compounded manufacture of noodles is to increase the foods’ nutritious content, and it can result in increased cooking losses and decreased flexibility because of the reduction of gluten (19, 20). Colored wheat varieties present a promising solution to this challenge, as they provide gluten levels comparable to conventional wheat flour (9). This gap necessitates investigation to leverage colored wheat in developing nutritionally enhanced, high-quality noodles without sacrificing critical processing attributes.

However, a systematic comparison of the three major colored wheat types (blue, purple, and black), particularly regarding their performance in noodle-making and their ability to retain bioactive compounds during boiling, is still lacking. Such a comparison is crucial to identify the most suitable colored wheat variety for specific nutritional and quality objectives in staple food applications.

This study therefore aimed to conduct a systematic comparative evaluation of noodles prepared from the three predominant types of colored whole wheat (blue, purple, and black) against a white whole wheat baseline. We comprehensively investigated the farinographic properties of the flours, the cooking quality, textural attributes, and microstructure of the noodles, and critically assessed the retention of anthocyanins and antioxidant capacity throughout processing and cooking. The findings are expected to elucidate the distinct advantages and limitations of each colored wheat variety, providing crucial insights for developing nutritionally fortified noodle products tailored to specific functional attributes.

## 2 Materials and methods

### 2.1 Materials

Four wheat cultivars (cv.) with different cereal colors namely cv. Jimai 22-White grain (W), cv. Yuzhoulanmai 1 hao-Blue grain (B), Yuzhouheimai 1 hao-Purple grain (P), cv. Yuzhouzimai 3 hao-Black grain (Bl) were used in this study. All of the samples were provided by the College of Agronomy at Henan Agricultural University.

### 2.2 Sample preparation

#### 2.2.1 Preparation of whole wheat flour

Wheat with different colors was cleaned and then milled into flour through a cyclone mill (JXFM110, Daji photoelectric Instrument Co., Ltd., Hangzhou, China) to obtain whole wheat flour (Figure S1).

#### 2.2.2 Preparation of noodles

The proportions of colored wheat flour to white wheat flour were 100, 75, 50, 25, and 0%, respectively (Table 1). The flour was mixed and blended in the flour mixer (JHMZ 200, Dongfu Jiuheng Instrument Technology Co., LTD., Beijing China) with purified water to create dough with the desirable consistency. The proofed dough was sheeted, pressed to 3.5 mm, and progressively rolled to approximately 1 mm thickness, with folding and sheeting repeated three times for subsequent testing. The dough sheet was precisely cut into noodles with a thickness of 1 mm and a width of 2 mm by cutting blades with a diameter of 2 mm. The dried noodles were made through four steps of cold air fixing, tide sweating, temperature rising and tide lowering.

**Table 1:** The designation of noodles in different proportions.

Number	Whole wheat flour composition		Name
1	White grain wheat 100%	–	W-100
2	White grain wheat 75%	Blue grain wheat 25%	B-25
3	White grain wheat 50%	Blue grain wheat 50%	B-50
4	White grain wheat 25%	Blue grain wheat 75%	B-75
5	–	Blue grain wheat 100%	B-100
6	White grain wheat 75%	Purple grain wheat 25%	P-25
7	White grain wheat 50%	Purple grain wheat 50%	P-50
8	White grain wheat 25%	Purple grain wheat 75%	P-75
9	–	Purple grain wheat 100%	P-100
10	White grain wheat 75%	Black grain wheat 25%	Bl-25
11	White grain wheat 50%	Black grain wheat 50%	Bl-50
12	White grain wheat 25%	Black grain wheat 75%	Bl-75
13	–	Black grain wheat 100%	Bl-100

### 2.3 Chemical composition

The moisture content of colored whole wheat flour was determined following the AOAC 990.20 method. Concurrently, the ash content was determined in accordance with AOAC 923.03. Protein content was assessed using the methodology of AOAC 991.20, and the starch content was evaluated with reference to AOAC 996.11. Dietary fiber content was analyzed using the AOAC 2009.01 method. Furthermore, the fat content was determined adhering to the method of AOAC 945.16, and the mineral content was determined through the application of AOAC 999.14. Color values (L*, a*, and b*) were measured using a portable colorimeter (NR110, Shenzhen 3NH Technology Co., Ltd., Shenzhen, China) based on the CIE L* a* b* system.

### 2.4 Anthocyanin content and antioxidant properties

Sample preparation: dried noodle samples were ground into powder. Cooked noodles were frozen at −18 °C for 12 h, freeze-dried using a vacuum freeze-dryer, and subsequently ground. All powders were sieved through an 80-mesh sieve.

#### 2.4.1 Total anthocyanin content (TAC)

The total anthocyanin content was determined by slightly modifying the method of Park et al. (21). Acidified methanol (methanol: 1.0 M HCl = 85:15 (v/v), pH = 1.0) was used to extract anthocyanins from sample. Continuous extraction was performed for 30 min using a water-bathing constant temperature vibrator (200 rpm, 50 °C) and the sample was shaken every 10 min to ensure full extraction. Whole wheat flour and dried noodles were extracted twice, respectively, with a material-to-liquid ratio of 1:10 and 1:5. Cooked noodles were extracted once with a material-to-liquid ratio of 1:10. After centrifugation at 10,000 rpm for 5 min, the supernatant was collected. Absorbance was measured at 535 nm using a UV–Vis spectrophotometer, with acidified methanol as the blank reference. Data were expressed as milligrams of cyanidin-3-O-glucoside per 100 grams of sample.

#### 2.4.2 Antioxidant activities

The ABTS radical-scavenging activity of whole wheat flour and thirteen noodle types was determined before and after cooking according to Multescu et al. (22) with slight modifications.

Total phenolics extracts: the sample is added methanol according to the ratio of 1:10, and then stored at room temperature 30 min in the dark. The extraction was centrifuged at 8000 rpm for 10 min. Next, add the methanol at a ratio of 1:5, extract it again using the same procedure, and then mix the supernatant extracted twice.

The total phenolics extract (0.4 mL) and diluted ABTS working solution (3 mL) were combined in a test tube, manually shaken thoroughly, and incubated in darkness for 30 min. Absorbance was measured using an ultraviolet spectrometer (UV-2000, Uniko Instrument Co., Ltd., Shanghai, China). A mixture of 0.4 mL methanol and 3 mL diluted ABTS working solution served as the blank, and distilled water was used as the reference. Results are expressed as milligrams of ascorbic acid per gram of noodle and calculated according to the following Equation 1:


(1) $$\text{Percentage inhibition}\;(\%)=\frac{\left(A_{1}-A_{0}\right)}{A_{0}}\times 100\%$$

Where: *A_1_* = absorbance after adding the sample solution. *A_0_* = absorbance of the blank solution.

### 2.5 Farinographic properties

Farinographic properties were tested using a Frinograph-AT equipped with a 50 g bowl (Brabender, Duisburg, Germany) with the method described by Ghanate and Annapure (23). A flour sample was placed in a stainless steel bowl of the flour meter. A certain amount of distilled water was calculated from the water content of the sample recommended by the instrument and added to the bowl within 20 s to form the dough. As the dough was mixed, a curve was recorded by the flour meter on the chart paper, measuring parameters such as Water Absorption (WA), Dough development time (DDT), Stability Time (ST), Degree of Softening (DS) and Farinograph Quality Number (FQN).

### 2.6 Cooking properties

#### 2.6.1 Optimum cooking time (OCT)

A 10 g sample of noodles (15 cm in length) was cooked in 500 mL of boiling water (maintained at 1200 W). After 1 min, noodles were removed every 10 s, compressed between two glass plates, and examined for the presence of a white core. The time at which the white core completely disappeared was recorded as the optimum cooking time.

#### 2.6.2 Cooking loss

Noodles cooked to optimal time were drained, blotted with absorbent paper to remove excess surface water, and weighed after approximately 5 min. The remaining noodle soup was maintained at 500 mL; a 40 mL aliquot was transferred to an aluminum box and dried in an air oven at 105 °C for 12 h to constant weight. The dry matter loss rate and water absorption rate of the noodles were calculated according to the following Equation 2:


(2) $$\text{Cooking loss}\;(\%)=\frac{m_{1}}{m_{0}(1-w)}-\frac{v_{0}}{v_{1}}\times 100\%$$

Where: *m_0_* = mass of strips before steaming (g), *m_1_* = mass of 40 mL of noodle broth after drying (g), *w* = moisture (%) of steaming, *v*_0_ = volume of noodle broth fixed (mL), *v*_1_ = volume of broth weighed (mL).

### 2.7 Textural properties

The texture profile of cooked noodles was analyzed using a TA-XT2i texture analyzer (Stable Micro Systems, UK) according to the method of Xu (24) with slight modifications. The noodle samples were cooked in 500 mL of boiling water until reaching the optimum cooking time. Immediately after cooking, the noodles were rinsed with distilled water for 30 s. Six parallel groups of noodles were evaluated within 5 min after cooling. The noodles were arranged on a flat metal plate and measured using a cylindrical probe (P/36R, 36 mm). TPA was set to a pretest speed of 2.0 mm/s, test speed of 0.8 mm/s, post test speed of 2.0 mm/s, strain of 75%, and interval time of 1 s.

### 2.8 Scanning Electron microscope

The surface and section profile of frozen cooked noodles with different ratios were analysed using Scanning Electron Microscope (SEM) (S4800, Hitachi, Tokyo, Japan), based on the method of Lei (25) with slight modifications. The different prepared noodles were prefrozen at −18 °C and then vacuum freeze-dried and broken with tweezers to expose the broken cross section. The noodle samples with the broken cross section were glued to the specimen stage of the electron microscope for gold spraying treatment, and then placed under the scanning electron microscope to observe the microstructure of the broken section of the cooked noodles. The images were acquired at an accelerating voltage of 3.0 kV with magnifications of 150.

### 2.9 Statistical analysis

Data were expressed as mean ± standard deviation and were subjected to one-way analysis of variance (ANOVA) followed by Tukey’s multiple comparisons test using GraphPad Prism 9 (GraphPad Software, CA, USA), with significance defined at *p* < 0.05.

## 3 Results and discussion

### 3.1 Qualitative of different wheat flours

#### 3.1.1 Comparative analysis among colored and white wheat flours

The protein content played a crucial role in the cooking quality of noodle products. Table 2 listed the fundamental components of the flours, including their protein and dietary fiber contents. All four kings of whole wheat flour are high GPC (Grain protein content, >12%) wheat varieties, which have great potential in providing nutrition-fortified wheat diets for many social groups and improving the export potential of wheat reported by Padhy et al. (26). The protein content of colored whole wheat flour was significantly (*p* < 0.05) higher than that of white whole wheat flour, and blue whole wheat flour had the highest protein content. This suggested that substituting these three colored whole wheat flours for white whole wheat flour might greatly increase the protein content of the final product. There was no significant difference in the starch and dietary fiber content among the four kinds of whole wheat flours; the ash content of black whole wheat flour was significantly (*p* < 0.05) higher than that of the other three flours; the fat content of purple whole wheat flour and black whole wheat flour was significantly (*p* < 0.05) higher than that of the other two flours.

**Table 2:** Chemical composition of whole wheat flour with different grain colors.

Sample	Protein (%)	Starch (%)	Dietary fiber (%)	Ash (%)	Fat (%)
W	12.69 ± 0.08^c^	60.82 ± 0.68^a^	11.96 ± 0.16^a^	1.71 ± 0.00^b^	1.68 ± 0.04^b^
B	14.00 ± 0.08^a^	58.40 ± 1.76^a^	10.88 ± 0.34^a^	1.64 ± 0.02^bc^	1.33 ± 0.02^b^
P	13.62 ± 0.04^b^	60.92 ± 0.76^a^	11.90 ± 0.24^a^	1.52 ± 0.04^c^	3.04 ± 0.14^a^
Bl	13.63 ± 0.01^b^	60.44 ± 0.28^a^	11.18 ± 0.32^a^	2.13 ± 0.04^a^	2.94 ± 0.18^a^

Data are presented as mean values ± standard deviations (SD) of independent experiments (*n* = 3). The values designated by the different small letters in the columns of the table are significantly different (*p* < 0.05).

**Figure 1:**
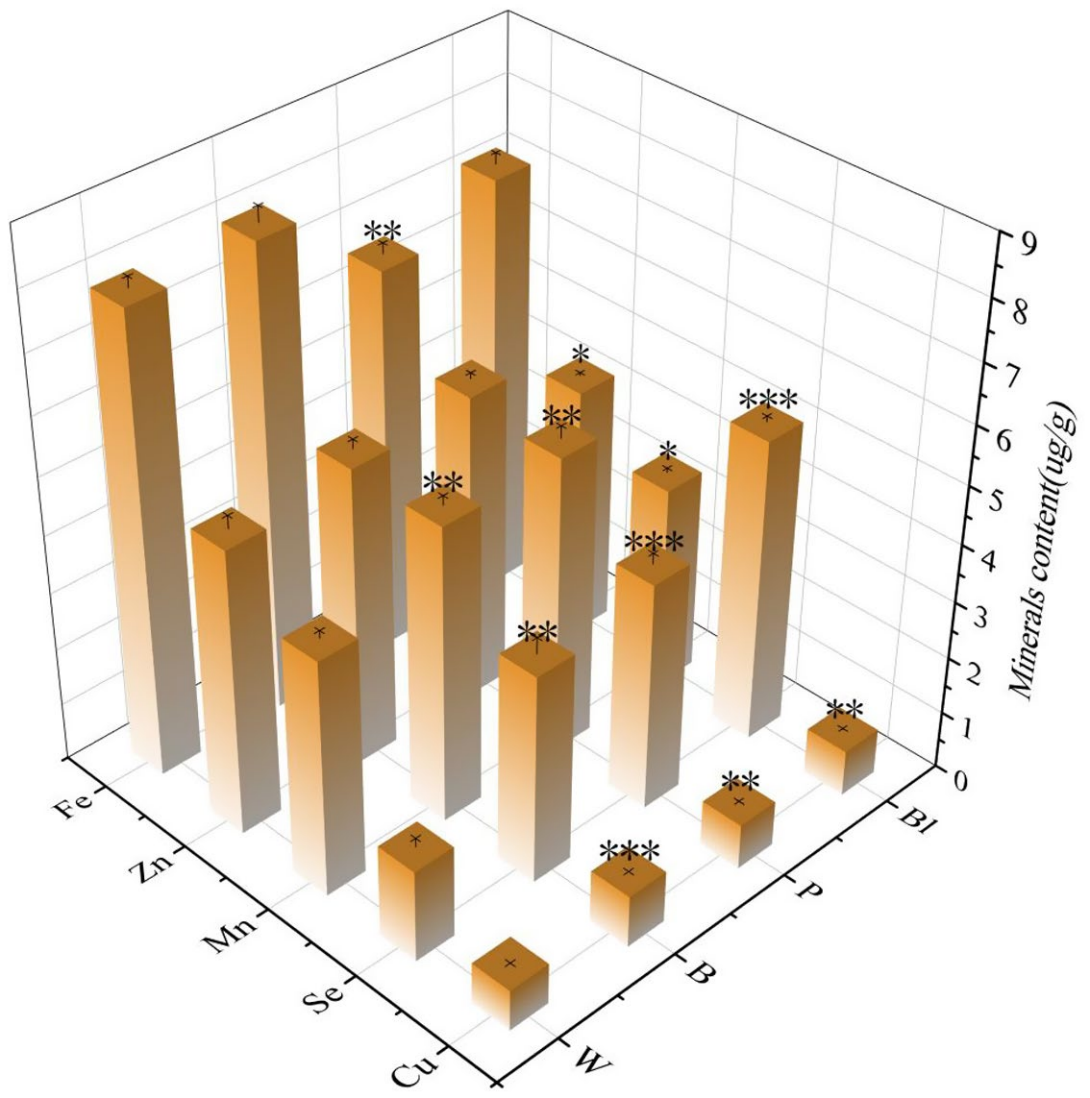
Minerals content of the whole wheat flour with different grain colors. *, **, *** denote that different colored wheat flours of the same mineral show marked differences (*p* < 0.05, *p* < 0.01, *p* < 0.001) compared to whole white wheat flour.

Fe, Mn, Zn, Cu, and Se are essential trace elements for human health, with wheat serving as a vital dietary source of these elements. Mineral elements are abundant in all four kinds of whole wheat flour (Figure 1). Specifically, the colored whole wheat flours had significantly (*p* < 0.05) higher concentrations of Cu (0.72 μg/g - 0.91 μg/g) and Se (1.61 μg/g - 5.28 μg/g) than those of white whole wheat flour, with the Se content increasing by more than twofold. This substantial elevation, particularly the graded increase observed among the colored wheat types, probably suggests a potential nutritional advantage associated with wheat pigmentation.

#### 3.1.2 Differences in dough behavior between colored and white wheat flours

Farinographic tests provide useful information to assess dough behavior during the mixing phase. DDT, ST, and FQN were indicators that reflected the compactness of the gluten network and the strength of the dough. The higher these values were, the firmer the dough became, and it exhibited greater resistance to shear forces (27). Higher DS values, reflecting reduced dough resistance to mechanical stress during mixing, indicated greater susceptibility to deformation, adversely affecting dough suitability for shaping during processing. Table 3 presents the farinographic properties of doughs prepared from four whole wheat flour samples. The water absorption of these flours (ranging from 77.07 to 79.40%) was significantly higher than the 58% reported for refined wheat flour by Awolu, Sudha, & Manohar (28). This elevated absorption is primarily due to the high bran content in whole wheat flour, which possesses enhanced water retention properties. For whole wheat flour, the ST range is 2.05 to 3.67 min, while the DDT range is 3.00 to 4.30 min. Blue and purple whole wheat flours showed significantly lower DDT and ST values, suggesting potential impairment of the gluten network structural integrity. The ST of purple whole wheat flour was the lowest, and its DS was the highest (162.67 FU). One probable reason is that it has a significantly higher fat content (Table 2). Protein and water can be separated by fat, which prevents gluten from forming. Similarly, Mu et al. (29) reported decreased DDT and ST along with increased DS in flours with reduced gluten content. The DDT and ST of white and black whole wheat flours are the longest, indicating that the doughs are firmer and more tenacious, and therefore have a higher FQN. It is clear that black whole wheat flour has a wide range of opportunities to replace white whole wheat flour in the manufacturing of wheat-based products because there are basically no significant differences between flour quality characteristics.

**Table 3:** Farinographic properties of whole wheat flour with different grain colors.

Sample	WA (%)	DDT (min)	ST (min)	DS (FU)	FQN (mm)
W	79.40 ± 0.14^a^	4.30 ± 0.18^a^	3.43 ± 0.08^b^	94.50 ± 2.12^d^	66.00 ± 0.00^a^
B	77.07 ± 0.32^b^	3.00 ± 0.11^c^	2.37 ± 0.09^c^	134.33 ± 6.51^b^	42.67 ± 2.08^b^
P	77.67 ± 0.12^b^	3.00 ± 0.04^c^	2.05 ± 0.09^d^	162.67 ± 7.37^a^	40.33 ± 1.53^b^
Bl	79.00 ± 0.30^a^	3.69 ± 0.16^b^	3.67 ± 0.10^a^	112.67 ± 3.06^c^	62.33 ± 1.53^a^

Data are mean ± SD of three replicates (*n* = 3). Means in the same column with different lowercase superscripts differ significantly (*p* < 0.05). WA, water absorption; DDT, dough development time; ST, stability time; DS, degree of softening; FQN, farinograph quality number.

### 3.2 Noodles quality

#### 3.2.1 Color

Dried noodle color is an important quality attribute that influences consumers. The variations of noodles in appearance, color, and inherent components can be specifically described using the L*, a*, and b* values. Upon addition of colored wheat flour, the noodles showed an obvious color change that gradually varied with the color of the wheat grain (Table 4). The obvious color difference among the noodle groups might be due to the presence of phenolic compounds like anthocyanins brought by the addition of colored whole wheat flour. The L* values of colored wheat flour and noodles were significantly lower than those of white wheat, indicating a darker appearance. Purple whole wheat flour and black whole wheat flour exhibited the lowest L* values (83.20 and 83.91, respectively), with a similar trend observed in their corresponding noodles. In contrast, the a* values of purple whole wheat flour and black whole wheat flour (5.28 and 4.77, respectively) and their corresponding noodles (ranging from 6.34 to 8.00), were higher, indicating a more noticeable redness component. These results demonstrate, as discussed in the previous study, that the significant color differences observed in the noodles are primarily driven by the anthocyanin content inherent in the colored whole wheat flours (5). The total color change (∆E) was calculated by comparing with the colour parameters of the white standard plate. For the flours, this parameter (∆E) showed no significant difference between blue whole wheat flour and white whole wheat flour. In contrast, ∆E values significantly increased (*p* < 0.05) in noodles prepared with blue whole wheat flour. This suggests that thermal-mechanical processing likely induced the release of bound anthocyanins and matrix homogenization, thus making color changes more pronounced.

**Table 4:** Effect of colored wheat on the color changes of whole wheat flour, dried noodles.

Sample		L*	a*	b*	△E
Whole wheat flour	W	88.79 ± 0.06^a^	4.75 ± 0.01^b^	10.75 ± 0.20^a^	16.24 ± 0.12^c^
	B	84.98 ± 0.62^b^	2.40 ± 0.01^c^	5.81 ± 0.22^d^	16.29 ± 0.49^c^
	P	83.20 ± 0.61^b^	5.28 ± 0.14^a^	8.19 ± 0.19^b^	19.42 ± 0.49^a^
	Bl	83.91 ± 0.26^b^	4.77 ± 0.17^b^	6.77 ± 0.13^c^	18.10 ± 0.25^b^
Control	W-100	71.22 ± 0.15^a^	5.34 ± 0.11^a^	15.30 ± 0.21^a^	33.03 ± 0.20^c^
Blue grain wheat	B-25	59.12 ± 0.41^c^	4.91 ± 0.02^b^	13.64 ± 0.35^b^	43.38 ± 0.49^a^
	B-50	62.07 ± 1.10^b^	2.62 ± 0.02^c^	11.94 ± 0.30^c^	39.86 ± 0.97^b^
	B-75	59.34 ± 0.99^c^	2.15 ± 0.04^d^	10.62 ± 0.17^d^	42.08 ± 0.91^ab^
	B-100	57.07 ± 1.38^c^	1.16 ± 0.05^e^	8.89 ± 0.44^e^	43.86 ± 1.27^a^
Purple grain wheat	P-25	60.42 ± 0.76^bc^	7.13 ± 0.30^b^	14.56 ± 0.16^b^	42.76 ± 0.74^b^
	P-50	62.69 ± 0.55^b^	6.34 ± 0.01^c^	12.55 ± 0.21^c^	39.87 ± 0.45^c^
	P-75	58.61 ± 1.62^c^	7.57 ± 0.35^ab^	12.19 ± 0.19^c^	43.82 ± 1.55^b^
	P-100	55.51 ± 0.37^d^	7.82 ± 0.24^a^	11.06 ± 0.33^d^	46.50 ± 0.25^a^
Black grain wheat	Bl-25	59.92 ± 0.37^b^	7.42 ± 0.21^b^	13.63 ± 0.42^b^	42.98 ± 0.36^b^
	Bl-50	55.00 ± 1.07^c^	8.00 ± 0.02^a^	11.79 ± 0.36^c^	47.21 ± 0.93^a^
	Bl-75	53.22 ± 0.76^c^	7.76 ± 0.09^a^	10.40 ± 0.24^d^	48.55 ± 0.69^a^
	Bl-100	53.87 ± 0.70^c^	7.38 ± 0.06^b^	9.86 ± 0.23^d^	47.75 ± 0.66^a^

Data are mean ± SD of three replicates (*n* = 3). Different superscript lowercase letters (a-c) in the table indicate significant differences between groups (*p* < 0.05). L*: the brightness. a*: the red-greenness. b*: the yellow-blueness. △E: the total color difference.

#### 3.2.2 Textural profile of noodles

##### 3.2.2.1 Changes in cooking properties

Moisture content critically determines the shelf-life stability and textural quality of dried noodles. All noodle samples maintained moisture contents of 9.15–11.36% (Table 5), within the safe range for dried noodles established by Biernacka et al. (30). Notably, control noodles (W-100) generally exhibited lower moisture than their counterparts containing colored wheat flour. When the addition amount of colored wheat was increased from 25 to 100%, the moisture content of all noodle samples showed a decreasing trend within the suitable range. As an example, the moisture content decreased from 11.36% (B-25) to 9.15% (B-100).

**Table 5:** The results of qualities characterization for different types of noodles.

Sample		Moisture (%)	Cooking loss (%)	Hardness (g)	Adhesiveness (g·sec)	Springiness	Chewiness
Control	W-100	9.405 ± 0.082^c^	5.755 ± 0.230^b^	7058.620 ± 59.849^a^	−67.577 ± 1.152^d^	0.938 ± 0.002^b^	4650.119 ± 60.555^a^
Blue grain wheat	B-25	11.362 ± 0.247^a^	7.897 ± 0.532^a^	6123.708 ± 24.955^c^	−81.159 ± 3.803^c^	0.937 ± 0.001^b^	3636.053 ± 61.224^c^
	B-50	10.546 ± 0.098^b^	6.356 ± 0.599^ab^	6067.715 ± 119.715^c^	−94.745 ± 3.252^b^	0.935 ± 0.004^bc^	3998.384 ± 71.050^b^
	B-75	10.866 ± 0.022^ab^	8.338 ± 0.562^a^	6358.626 ± 35.313^b^	−94.570 ± 4.845^b^	0.944 ± 0.002^a^	3722.726 ± 39.798^c^
	B-100	9.148 ± 0.049^c^	7.576 ± 0.510^ab^	6480.377 ± 68.269^b^	−104.085 ± 2.114^a^	0.929 ± 0.000^c^	3738.566 ± 16.481^c^
Purple grain wheat	P-25	9.405 ± 0.082^a^	5.755 ± 0.230^c^	6278.626 ± 61.550^b^	−104.646 ± 3.764^c^	0.947 ± 0.003^a^	4082.408 ± 49.935^b^
	P-50	10.376 ± 0.513^a^	7.499 ± 0.034^a^	6338.876 ± 38.451^b^	−110.583 ± 1.473^bc^	0.940 ± 0.002^ab^	3843.348 ± 79.735^b^
	P-75	10.053 ± 0.006^a^	5.217 ± 0.054^d^	5347.077 ± 104.439^d^	−116.669 ± 0.461^b^	0.910 ± 0.006^c^	2941.885 ± 64.880^c^
	P-100	10.199 ± 0.010^a^	6.534 ± 0.148^b^	5752.798 ± 67.282^c^	−160.869 ± 4.681^a^	0.886 ± 0.000^d^	2562.585 ± 310.129^c^
Black grain wheat	Bl-25	9.588 ± 0.249^a^	6.972 ± 0.039^b^	6751.360 ± 76.724^c^	−94.301 ± 3.532^a^	0.928 ± 0.001^c^	4332.460 ± 47.992^d^
	Bl-50	9.405 ± 0.082^b^	5.755 ± 0.230^c^	6436.049 ± 18.792^d^	−100.149 ± 6.947^a^	0.931 ± 0.001^bc^	4085.169 ± 16.226^e^
	Bl-75	11.175 ± 0.495^a^	7.564 ± 0.144^a^	7580.262 ± 47.165^a^	−97.047 ± 1.683^a^	0.935 ± 0.006^bc^	5214.600 ± 91.325^a^
	Bl-100	11.117 ± 0.231^a^	7.109 ± 0.374^ab^	6781.878 ± 100.560^c^	−93.771 ± 4.577^a^	0.944 ± 0.002^a^	4823.702 ± 20.589^b^

Data are presented as mean values ± SD of independent experiments (*n* = 3). Different superscript lowercase letters (a-e) in the table indicate significant differences between groups (*p* < 0.05).

Cooking loss is a pivotal factor in assessing noodle quality, indicating the amount of solid content leached from noodles into cooking water. Tan, Tan, & Easa (18) reported that noodles achieve favorable sensory scores when cooking loss is below 10%. In this study, all noodle samples (5.22–7.90%) exhibited cooking losses below this threshold, demonstrating acceptable cooking properties for consumers in formulations with added colored wheat. Furthermore, noodles containing colored wheat flour typically exhibit higher cooking loss than W-100 (Table 5). This may result from reduced cross-linking density in the gluten network of formulations with added colored wheat, weakening the structure and accelerating starch leaching and solid loss, thereby increasing cooking loss (31).

##### 3.2.2.2 Textural properties

The texture of cooked noodles, characterized by its textural properties, is a critical determinant of the eating quality in dried noodle products. As shown in Table 5, the hardness and chewiness of noodles added with colored whole wheat flour were generally lower than those of W-100, while the adhesiveness became higher. This phenomenon may be attributed to increased starch leaching during cooking, which elevates the paste viscosity of colored wheat noodles, while matrix disintegration reduces hardness and chewiness. Xu et al. (32) found that the hardness of noodles was positively correlated with their chewiness and negatively correlated with their adhesiveness. Moreover, the likely higher content of mechanically damaged starch granules in colored wheat may results in extreme water absorption capacity, which leads to rapid elevation of adhesiveness (33). However, Bl-100 and Bl-75 exhibited distinct textural properties from other composite noodles, closely resembling W-100. This further demonstrates that black whole wheat flour possesses superior functionality for diverse flour-based products compared to other colored wheat varieties. We observed that P-100 displayed the lowest chewiness and P-75 the lowest hardness. This aligns with the lower ST and higher DS of purple wheat, which can impede gluten development and reduce structural compactness, resulting in diminished noodle chewiness. Interestingly, the addition of colored wheat had basically no significant effect on the springiness of the noodles (the speed at which the noodles rebounded under the action of external force).

#### 3.2.3 Scanning Electron microscope

**Figure 2:**
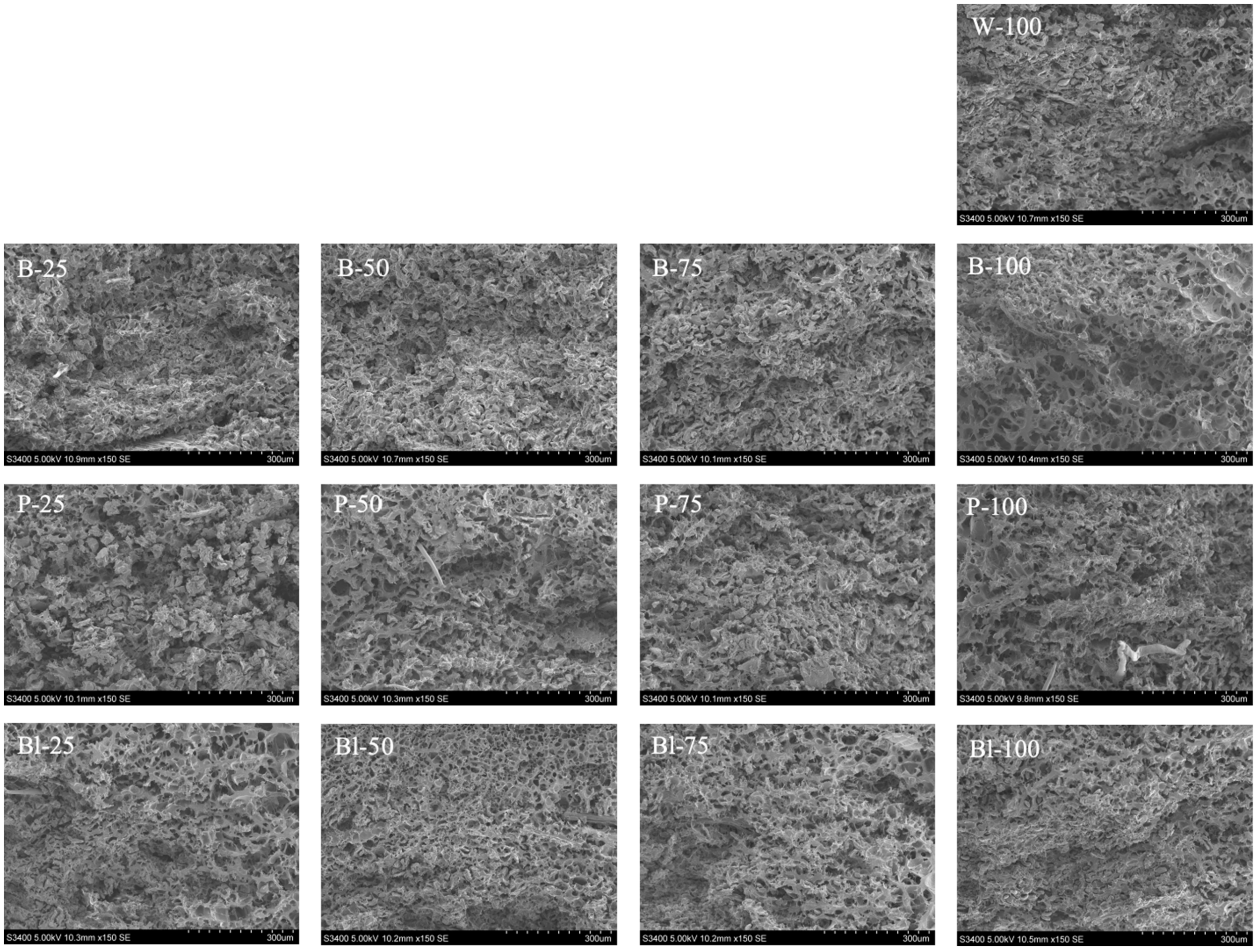
Scanning electron micrographs of cooked noodles prepared with different ratio of flour.

The microstructure of noodles is a critical determinant of their quality. As shown in Figure 2, the cross-section microstructures of noodles with different ingredient ratios were observed under Scanning Electron Microscope at 150 magnifications. Scanning electron microscopy indicated that the incorporation of colored wheat flour compromised gluten cross-linking in the noodles. This was likely due to reduced structural continuity and uniformity, more distinct gluten-starch boundaries, and increased pore formation. There was a certain impact of anthocyanin on the gluten cross-linking process. When the content of anthocyanin was greater than 0.3%, the voids in the gluten structure became larger and the gluten network became looser (34). Compared to W-100, the content of anthocyanins in colored noodles has increased by 55.95–82.50%. Excessive increase of anthocyanins might disrupt the gluten network structure, leading to an increase in cooking loss and a decrease in hardness of the noodles (Table 5). According to Zhang et al. (20) and Farzana et al. (19), noodles with weak structure had a higher cooking loss. Interestingly, although the variation in anthocyanin content in the noodles is significant, starch was well encapsulated in the internal structure, and the cooking loss was also within an acceptable range, which indicates that the addition of colored whole wheat flour has a positive impact on the noodle products. The different encapsulation conditions of starch granules in whole wheat flour noodles were related to the density of the gluten network structure (35). Guo et al. (36) suggested that the interaction between protein and anthocyanin was beneficial to the quality of noodle products.

### 3.3 Processing effects on anthocyanin and antioxidant properties

#### 3.3.1 Anthocyanin content

**Figure 3:**
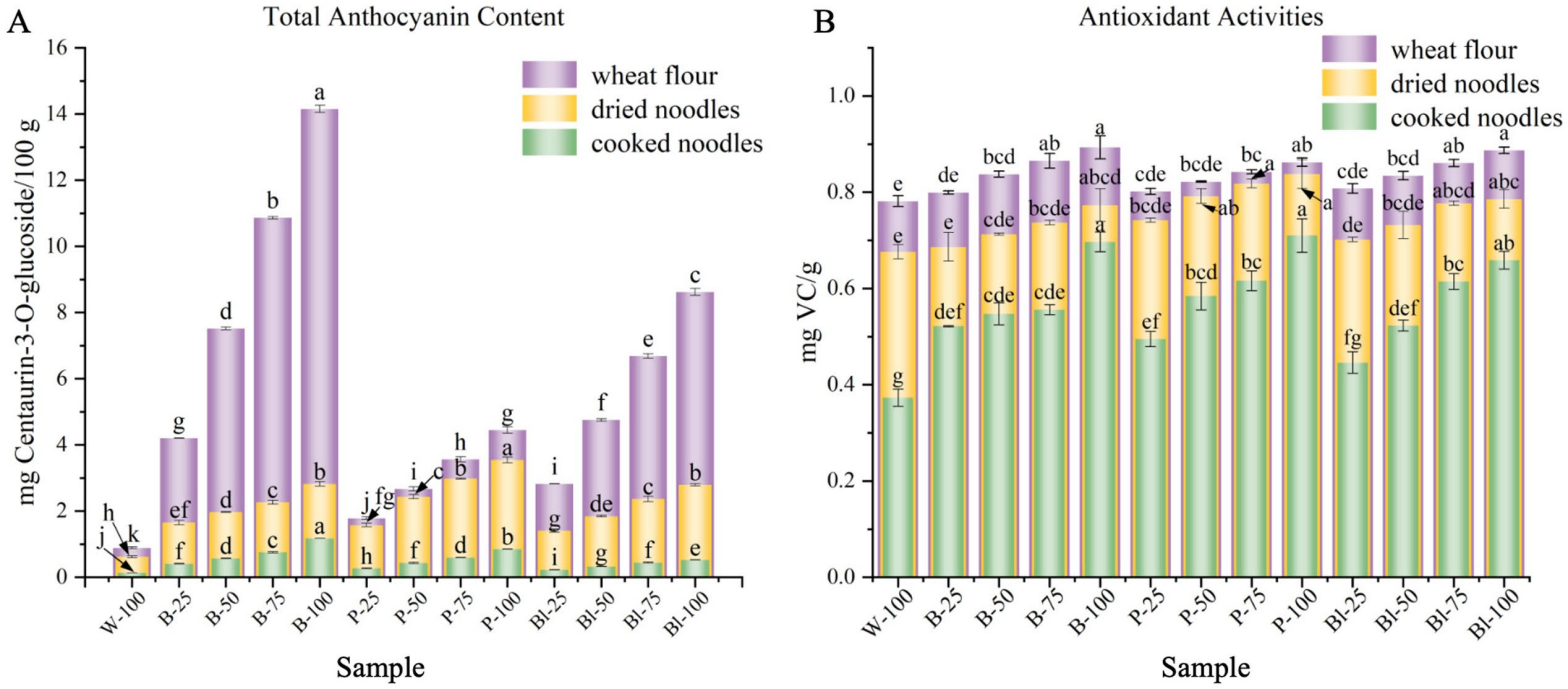
Comparison of anthocyanin content and antioxidant activity of whole wheat flour, dried noodles and cooked noodles. **(A)** total anthocyanin content; **(B)** antioxidant activity-ABTS. Different letters indicate significant differences at *p* < 0.05 between groups.

Anthocyanins, as remarkable antioxidants, have also been found to uphold antihypertension, antihyperglycemic, and anticancer (4, 6). Figure 3A showed the levels of TAC of the flours, dried noodles and cooked noodles. As Sytar et al. (6) has demonstrated, wheat grains of different colors have different total anthocyanin contents. The total anthocyanin content in whole wheat flour ranges from 0.89 mg/100 g to 14.15 mg/100 g. Compared with white whole wheat flour, higher total anthocyanin content levels were significantly (*p <* 0.05) observed in whole flours of colored wheat. Meanwhile, the dried noodles and cooked noodles obtained from these wheat lines also demonstrated high TAC (*p <* 0.05). Similar tendency was obtained by Pasqualone et al. (12) when purple wheat lines was added to biscuits.

Due to the complex processes experienced during the manufacturing process of dried noodles, it needs to be taken in account that a very significant reduction was presented from all variety whole wheat flours to whole wheat noodles, which was also reported by Ficco et al. (37). Conducting in-depth research to optimize the manufacturing process of fine dried noodles for better TAC retention rate would be an appropriate approach in the future. Additionally, the influences of cooking on the anthocyanin components were also observed. Similar rates of TAC reduction were observed in the noodles after cooking, which may be due to the diffusion of anthocyanin from the noodles into the soup and thermal degradation during the cooking process, but the TAC of all colored wheat varieties was still significantly (*p* < 0.05) higher than that of white wheat varieties. Similar findings were reported in different composite noodle products (38).

#### 3.3.2 Antioxidant capacity of ABTS

The total antioxidant capacity of the flours, raw noodles and noodles were measured using the ABTS assay (Figure 3B). The antioxidant capacity of colored whole wheat flour was significantly higher than that of white whole wheat flour. Similarly, the antioxidant capacity of noodles increased both before and after cooking with the amount of colored wheat flour added. Regarding whole wheat flour, when compared with purple whole wheat flour, black whole wheat flour and blue whole wheat flour, Figure 3A indicated that the last possessed a significantly higher anthocyanin content level, however, this failed to result in a distinct advantage regarding antioxidant capacity. This was likely due to the fact that the first two contained more Se (Figure 1) than others. The increase of Se content aided in increasing the total phenolic content and enhancing the antioxidant capacity within plants. A similar situation was reported by Bhadwal, Sharma, & Singh (39) in rice. The antioxidant capacity also experienced varying degrees of loss during the preparation and cooking processes of noodles, and the same trend was observed in the changes of anthocyanin content. Irrespective of wheat varieties and powder grinding methods, the antioxidant capacity was weakened by processes such as drying, rolling and cooking. Therefore, the antioxidant capacity of the final products was often lower than that of the corresponding flours (5, 40). Our findings showed that the cooking process had a greater impact than the noodle preparation process (including rolling and drying) when comparing white whole wheat flour with W-100, purple whole wheat flour with P-100, and black whole wheat flour with Bl-100. However, the antioxidant capacity contributed by colored wheat was still much higher than that of white wheat for the cooked noodles, which were the part that actually entered people’s bodies.

### 3.4 Pearson’s correlation coefficients

**Figure 4:**
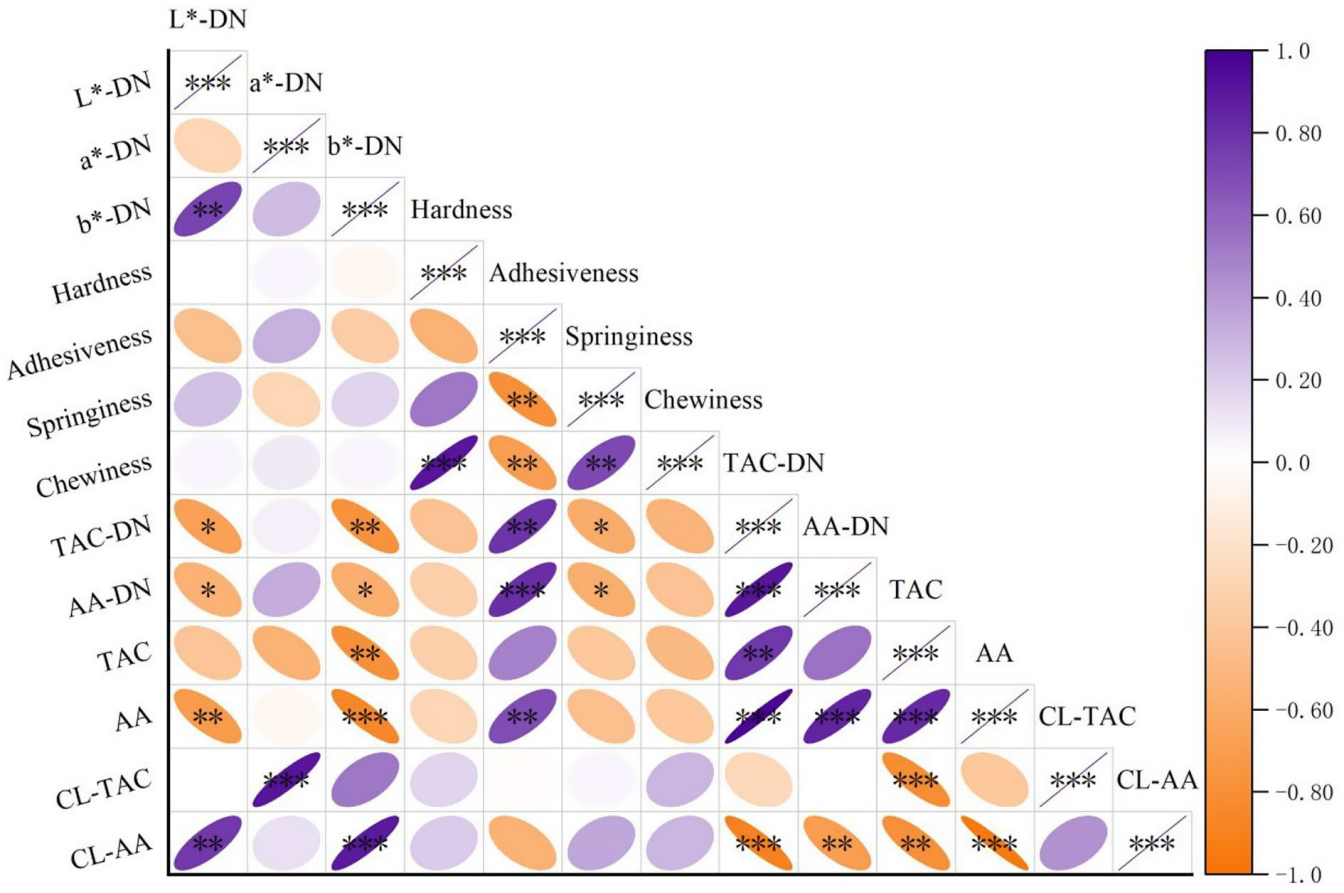
Correlation between the color changes, qualities characterization, anthocyanin content and antioxidant activity changes of noodles. DN, Dried noodles; TAC, Total anthocyanin content; AA, Antioxidant activity; CL, Cooking loss; The *p*-values are marked with asterisks (*, **, and ***) for *p* < 0.05, *p* < 0.01, *p* < 0.001.

Pearson’s correlation analysis was conducted to elucidate the relationships between key quality parameters across all noodle samples (Figure 4). Notably, when comparing the correlation patterns among the three colored wheat types, distinct varietal behaviors emerged, underscoring the necessity of a systematic comparative approach.

As anticipated, a strong negative correlation was observed between the TAC and color parameters (L* and b* values) (*p* < 0.01) across all colored varieties, confirming anthocyanins as the primary pigments responsible for the darker appearance (Figure S2). However, the strength of this correlation varied, being strongest in blue wheat noodles, which aligns with their highest anthocyanin concentration.

A highly significant positive correlation (*p* < 0.001) was identified between TAC and adhesiveness for the blue and purple wheat noodles, suggesting a direct impact of their specific anthocyanin profiles on stickiness. Intriguingly, this relationship was weaker in black wheat noodles, indicating that other components, such as its unique dietary fiber or protein matrix, might mitigate this effect and contribute to its superior textural properties closer to those of white wheat. Furthermore, a notable negative correlation (*p* < 0.01) was found between TAC and the loss of antioxidant activity during cooking (CL-AA). This protective effect against antioxidant loss was most pronounced in black wheat noodles. This suggests that the high selenium content in black wheat may play a synergistic role in stabilizing antioxidants during thermal processing, a phenomenon less evident in the blue and purple varieties (30, 36).

The correlation analysis not only confirmed the overall impact of colored wheat addition but also revealed fundamental differences in how each variety interacts with the noodle matrix. Blue wheat’s properties are predominantly driven by its high anthocyanin load, purple wheat shows an intermediate behavior, while black wheat often diverges, with selenium and other components imparting distinct technological and nutritional advantages that more closely resemble white wheat.

## 4 Conclusion

In conclusion, this study provides a systematic evaluation of noodles from blue, purple, and black whole wheat flours, revealing distinct varietal strengths for developing functional noodles. Black whole wheat flour is the standout candidate for high-percentage substitution (75–100%), combining the highest selenium content with farinographic properties and textural integrity comparable to white wheat. This makes it ideal for creating selenium-enriched staple noodles that meet daily dietary needs without compromising quality. Blue whole wheat flour, despite boasting the highest anthocyanin content, presents a trade-off; incorporation levels exceeding 50% led to increased cooking loss and reduced hardness. Purple whole wheat flour offers intermediate nutritional benefits but results in a softer texture.

The distinct attributes of these colored wheats allow for targeted product development to cater to diverse consumer preferences within the growing functional food market. Black wheat’s strong technological performance and micronutrient density make it suitable for mainstream, high-volume nutritious staple products. In contrast, the vivid colors and potent antioxidant properties of blue and purple wheat are key assets for creating visually distinctive and health-promoting niche products, best applied at moderate levels (50–75%) to balance biofunctional benefits with sensory acceptability.

## Data Availability

The datasets presented in this study can be found in online repositories. The names of the repository/repositories and accession number(s) can be found in the article/Supplementary material.
