# Supplementary material for: Quality and nutritional properties of noodles: a systematic comparison of noodles based on blue, purple, black, and white whole wheat flours
Source: Front Nutr. 2025 Dec 12;12:1738148. doi: 10.3389/fnut.2025.1738148 (PMC12742469; doi:10.3389/fnut.2025.1738148)
Supplement: Supplementary file 1 [file Data_Sheet_1.docx]

**Fig. S1.** Typical appearance of the whole wheat flour with different grain colors.

**Fig. S2.** Typical appearance of the different ratio of dried noodles.


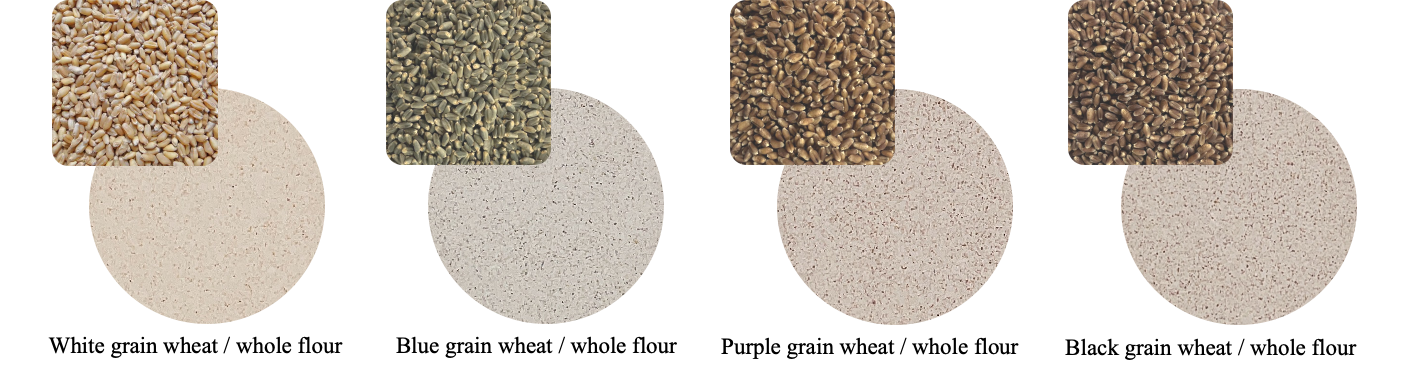


**Fig. S1**


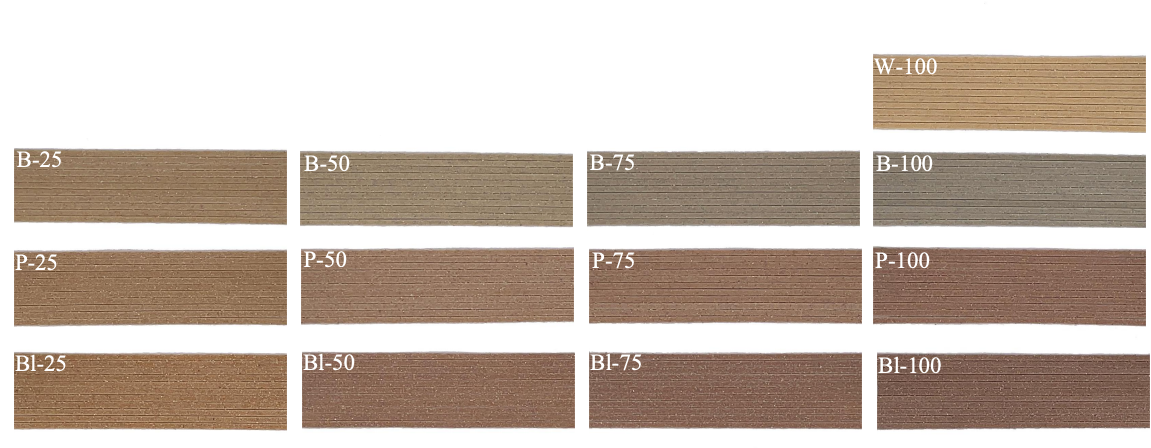


**Fig. S2**
